# Behavioural and social sciences for better health: call for papers

**DOI:** 10.2471/BLT.20.278481

**Published:** 2020-10-01

**Authors:** Elena Altieri, John Grove, Katrine Bach Habersaat, Susan Michie, Cass R Sunstein

**Affiliations:** a Department of Communications, World Health Organization, Avenue Appia 20, 1211 Geneva 27, Switzerland.; b Department of Quality Assurance, Norms and Standards, World Health Organization, Geneva, Switzerland.; c Vaccine-preventable Diseases and Immunization, World Health Organization Regional Office for Europe, Copenhagen, Denmark.; d Centre for Behavioural Change, University College London, London, England.; e Harvard Law School, Harvard University, Cambridge, United States of America.

What drives people towards tobacco use? What prevents people from doing physical exercise or adopting preventive measures during a pandemic? What increases the likelihood that someone will adhere to treatment or seek appropriate health care?

While a growing body of knowledge provides insights into these questions, factoring behavioural evidence effectively into health policies and programmes can be challenging. For example, health programmes often rest on the assumption that people will act in their best interest once we increase their awareness and knowledge. Yet, we know that this increase is insufficient for a behaviour to change.^1^ Because of inertia^2^ and preference for short-term rewards,^3^ people may continue with their unhealthy habits, despite improved health literacy. Certain health policies underestimate the importance of social norms and the fact that our behaviours are influenced by our perceptions of how other people think and act.^4^ Some interventions focus only on the human factor, without giving attention to environmental and structural issues that determine what options are available and how these options are presented to the population.

Too often, considerations around behaviours are only discussed in the implementation phase;^5^ but effective health policies and strategies require raising critical behavioural issues and questions much earlier, when broad policy objectives are discussed and designed.^6^ If we expect policy-makers and practitioners to increase the use of behavioural and social sciences, the global community of experts needs to provide easy access to evidence, tools, expertise and examples of use.

Behaviours are the result of complex interactions among cognitive, emotional, social and environmental drivers.^7^ To understand these, we need to draw theories and evidence from a variety of fields: sociology, behavioural sciences, behavioural economics, cognitive sciences, psychology, anthropology, humanities, communications, marketing, design thinking and system thinking.

To achieve health for all, policy-makers and practitioners need deeper insights into what shapes individual and collective behaviours^8^ among the general population as well as among practitioners and health-care workers who design and deliver health and social care.^9^ As part of its efforts to scale up the use of behavioural and social sciences in public health, the World Health Organization created a multidisciplinary technical advisory group for behavioural insights and sciences for health in 2020. The *Bulletin of the World Health Organization* will publish a theme issue on behavioural and social sciences for better health in 2021. We invite practitioners and researchers, particularly from low- and middle-income countries, to submit manuscripts with original research, reviews, perspectives and lessons from the field on the unique opportunities the behavioural and social sciences provide in achieving health for all.

We are interested in behaviourally informed approaches, interventions and reforms that have been shown to improve public health. In particular, we will welcome manuscripts that illustrate how behavioural sciences have been used for the design of policies and programmes; how behaviours of key players – including, but not only, at the population level – are addressed within health systems; how robust behavioural evidence can be gathered despite time and financial constraints; and how innovative approaches can help in overcoming these constraints. We hope submissions will provide evidence as to how multidisciplinary approaches improve the quality of behaviourally informed interventions; if and how existing behavioural theories and models are relevant to low- and middle-income countries; and how to build knowledge and skills relevant to behavioural and social sciences among health workers, practitioners and policy-makers.

The deadline for submissions is 31 December 2020. Manuscripts should be submitted in accordance with the *Bulletin*’s guidelines for contributors (available at: http://www.who.int/bulletin/volumes796/1/18-990118/en) and the cover letter should mention this call for papers.
